# Supplementary figures and images for: Dynamically expressed genes provide candidate viability biomarkers in a model coccidian
Source: PLoS One. 2021 Oct 1;16(10):e0258157. doi: 10.1371/journal.pone.0258157 (PMC8486141; doi:10.1371/journal.pone.0258157)

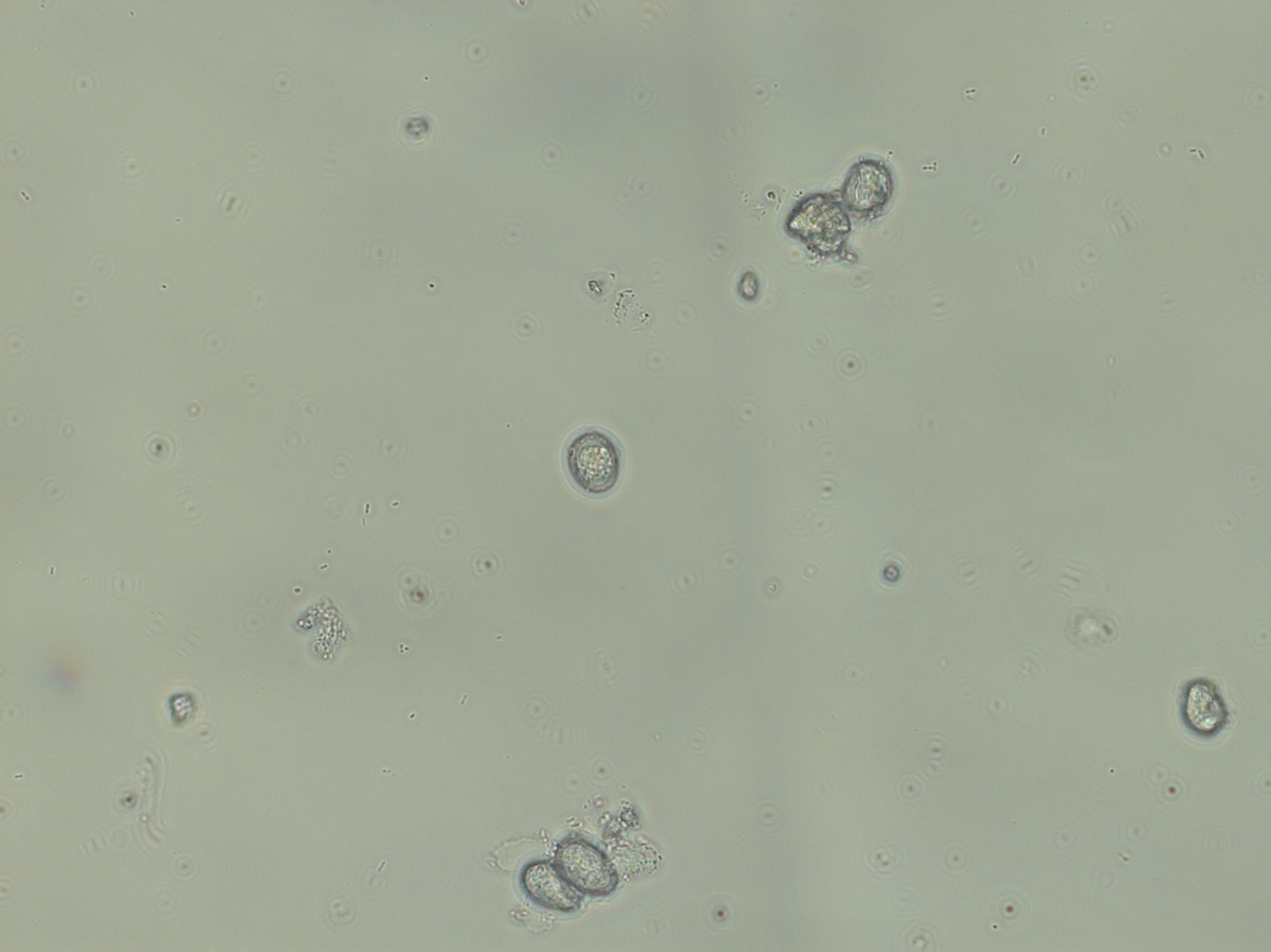

Supplement: S1 Fig — At hour 0, oocysts lack distinctive sporocysts. 100X under oil immersion. (TIF) [file pone.0258157.s001.tif]

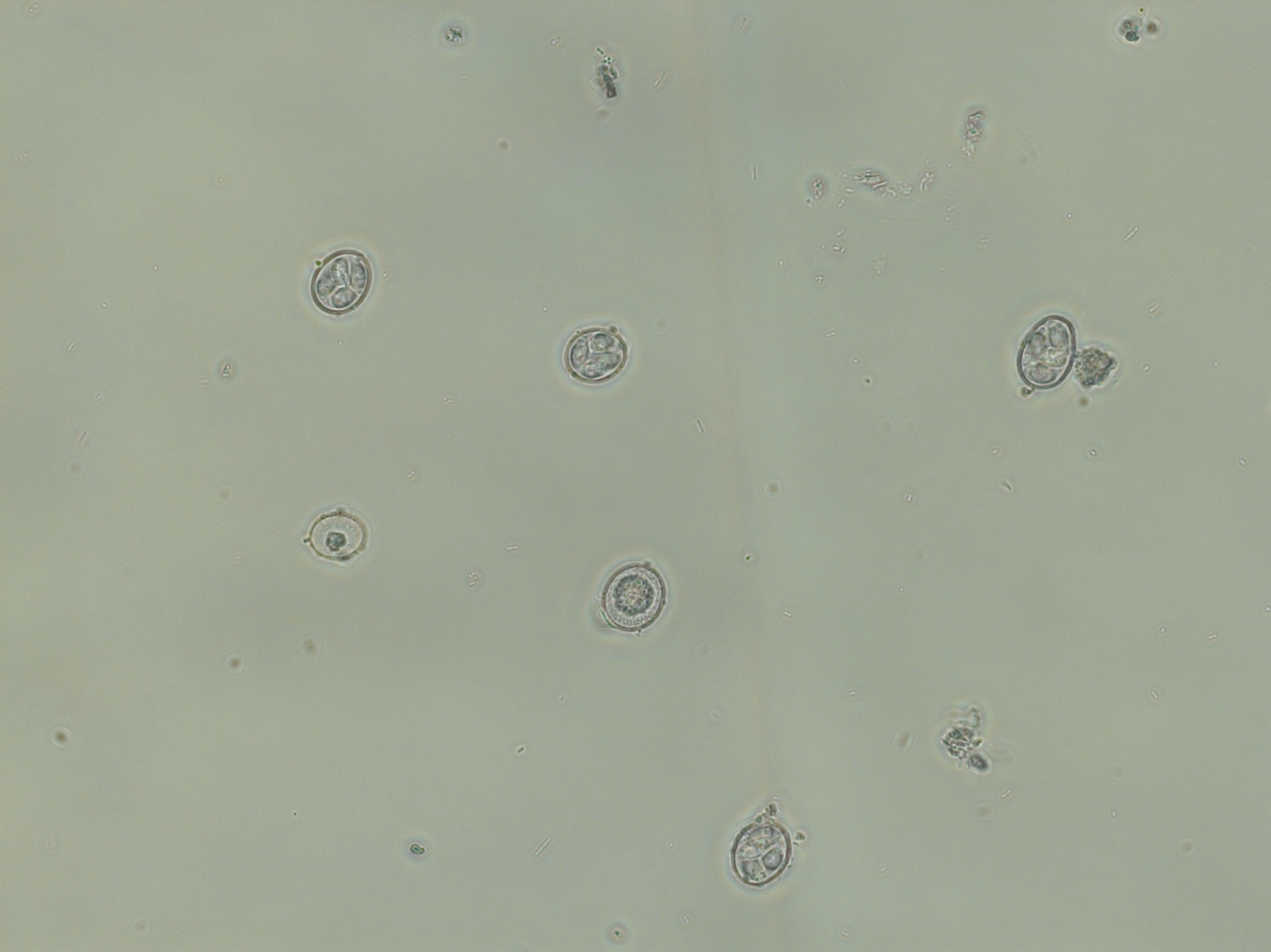

Supplement: S2 Fig — At hour 24, 83% of oocysts contain distinctive sporocysts. 100X under oil immersion. (TIF) [file pone.0258157.s002.tif]
