# Supplementary material for: Dynamically expressed genes provide candidate viability biomarkers in a model coccidian
Source: PLoS One. 2021 Oct 1;16(10):e0258157. doi: 10.1371/journal.pone.0258157 (PMC8486141; doi:10.1371/journal.pone.0258157)
Supplement: Attachment — Submitted filename: response to reviewers- Dynamic expression.docx [file pone.0258157.s015.docx]

September 14, 2021

We would like to thank you and the reviewers for your careful and constructive review, and are pleased to delineate our responses. To guide you, we structure our responses thus:

Editor and reviewer comments appear in blue.

*Brief explanatory comments appear in italics.*

Revised text is indented.

Please ensure that your manuscript meets PLOS ONE's style requirements, including those for file naming. The PLOS ONE style templates can be found at 
[https://journals.plos.org/plosone/s/file?id=wjVg/PLOSOne_formatting_sample_main_body.pdf](https://gcc02.safelinks.protection.outlook.com/?url=https%3A%2F%2Furldefense.com%2Fv3%2F__https%3A%2Fgcc02.safelinks.protection.outlook.com%2F%3Furl%3Dhttps*3A*2F*2Fjournals.plos.org*2Fplosone*2Fs*2Ffile*3Fid*3DwjVg*2FPLOSOne_formatting_sample_main_body.pdf%26data%3D04*7C01*7C*7C4b7a6c3f404e43c045ee08d96ce0fb67*7Ced5b36e701ee4ebc867ee03cfa0d4697*7C0*7C0*7C637660535835138555*7CUnknown*7CTWFpbGZsb3d8eyJWIjoiMC4wLjAwMDAiLCJQIjoiV2luMzIiLCJBTiI6Ik1haWwiLCJXVCI6Mn0*3D*7C1000%26sdata%3DFi*2FqYCxSo87YA0tUB5KE9z4qgXwcddISLxezIahwH9s*3D%26reserved%3D0__%3BJSUlJSUlJSUlJSUlJSUlJSUlJSUlJQ!!IkmNjII!VdUnM5iWVSiuS-IBnuPFLMxkhaZl3izcHl95AtEutm9vmSgf7YsDVGjXvHHPJ5Ve%24&data=04%7C01%7C%7Cb8c35de56791466922fd08d96d8794d1%7Ced5b36e701ee4ebc867ee03cfa0d4697%7C0%7C0%7C637661252233804186%7CUnknown%7CTWFpbGZsb3d8eyJWIjoiMC4wLjAwMDAiLCJQIjoiV2luMzIiLCJBTiI6Ik1haWwiLCJXVCI6Mn0%3D%7C1000&sdata=OvcL%2BHTSuqGNgJR19OX%2BDJwNIdXS2aArRA2ZmcRfs00%3D&reserved=0) and 
[https://journals.plos.org/plosone/s/file?id=ba62/PLOSOne_formatting_sample_title_authors_affiliations.pdf](https://gcc02.safelinks.protection.outlook.com/?url=https%3A%2F%2Furldefense.com%2Fv3%2F__https%3A%2Fgcc02.safelinks.protection.outlook.com%2F%3Furl%3Dhttps*3A*2F*2Fjournals.plos.org*2Fplosone*2Fs*2Ffile*3Fid*3Dba62*2FPLOSOne_formatting_sample_title_authors_affiliations.pdf%26data%3D04*7C01*7C*7C4b7a6c3f404e43c045ee08d96ce0fb67*7Ced5b36e701ee4ebc867ee03cfa0d4697*7C0*7C0*7C637660535835138555*7CUnknown*7CTWFpbGZsb3d8eyJWIjoiMC4wLjAwMDAiLCJQIjoiV2luMzIiLCJBTiI6Ik1haWwiLCJXVCI6Mn0*3D*7C1000%26sdata%3De3qDg89aVy*2BtUs2LA9Dv*2FLtdmN3cfct0GGFFLD*2B*2FtYM*3D%26reserved%3D0__%3BJSUlJSUlJSUlJSUlJSUlJSUlJSUlJSUlJQ!!IkmNjII!VdUnM5iWVSiuS-IBnuPFLMxkhaZl3izcHl95AtEutm9vmSgf7YsDVGjXvEaWRu1D%24&data=04%7C01%7C%7Cb8c35de56791466922fd08d96d8794d1%7Ced5b36e701ee4ebc867ee03cfa0d4697%7C0%7C0%7C637661252233814144%7CUnknown%7CTWFpbGZsb3d8eyJWIjoiMC4wLjAwMDAiLCJQIjoiV2luMzIiLCJBTiI6Ik1haWwiLCJXVCI6Mn0%3D%7C1000&sdata=KcPG2H2lobqVLBlfWRNSDCWR636A%2BSOmJx69KWGq9TI%3D&reserved=0)

*Thank you. We have reviewed each style requirement and believe the manuscript conforms to each. We have updated the author affiliations to indicate Dr. Tucker’s new address.*

2. In your Methods section, please include a comment about the state of the animals following this research. Were they euthanized or housed for use in further research? If any animals were sacrificed by the authors, please include the method of euthanasia and describe any efforts that were undertaken to reduce animal suffering.

*Thank you for the opportunity to elaborate on our commitment to animal welfare. We have elaborated that section accordingly:*

Animal experiments were performed following the protocol (19-04) approved by the Beltsville Area Animal Use and Care Committee, United States Department of Agriculture. Chickens employed in this study exhibited no outward signs of severe disease over the course of the study. After the study’s conclusion, these chickens were euthanized and all efforts were made to minimize animal suffering.

3. Thank you for stating the following financial disclosure: 
[This work was supported by USDA Projects “Detection and Control of Foodborne Parasites for Food Safety” 8042-32000-113-00D and “Development of Control and Intervention Strategies for Avian Coccidiosis” 8042-32000-111-00-D.] Please state what role the funders took in the study.  If the funders had no role, please state: "The funders had no role in study design, data collection and analysis, decision to publish, or preparation of the manuscript."

If this statement is not correct you must amend it as needed. Please include this amended Role of Funder statement in your cover letter; we will change the online submission form on your behalf.

*Thank you for asking about how our USDA projects are funded and structured.*

The authors designed the study, collected the data, and wrote the manuscript, as established by USDA’s Directive on Scientific Integrity. The Agricultural Research Service’s Office of National Programs established project objectives, and the Office of Scientific Quality and Review employed an outside, anonymous panel of experts to vet the research proposal for scientific merit. USDA supervisory scientists reviewed the manuscript to safeguard scientific integrity intellectual property, national security, and to maximize research impact.

4. We note that you have stated that you will provide repository information for your data at acceptance. Should your manuscript be accepted for publication, we will hold it until you provide the relevant accession numbers or DOIs necessary to access your data. If you wish to make changes to your Data Availability statement, please describe these changes in your cover letter and we will update your Data Availability statement to reflect the information you provide.

*Thank you. The USDA is committed to such data transparency. As stated in our submission, “The sequencing data generated in the work have been deposited in NCBI’s Gene Expression Omnibus (GEO) and are accessible through accession number GSE178256. All data generated or analyzed during this study are included in this published article and its supporting information files.” Upon acceptance, we will ensure that accession becomes immediately accessible.*

5. Please amend either the title on the online submission form (via Edit Submission) or the title in the manuscript so that they are identical.

*We apologize for that discrepancy. We have harmonized this by amending the title in the manuscript to mirror the title in the online submission form*:

Dynamically expressed genes provide candidate viability biomarkers in a model coccidian.

6. Please review your reference list to ensure that it is complete and correct. If you have cited papers that have been retracted, please include the rationale for doing so in the manuscript text, or remove these references and replace them with relevant current references. Any changes to the reference list should be mentioned in the rebuttal letter that accompanies your revised manuscript. If you need to cite a retracted article, indicate the article’s retracted status in the References list and also include a citation and full reference for the retraction notice.

*Thank you. We have reviewed the reference list and believe it is complete and accurate. We are not aware that any cited paper has been retracted.*

Reviewer #1: In this manuscript authors report their findings from a genome wide RNA-Seq study done using Eimeria acervulina oocysts to determine the biological processes involve in oocyst maturation in this organism. They also propose E. acervuline as a surrogate for Cyclospora cayetanensis, a related apicomplexan causing human disease. The data reported in the manuscript may provide a framework for a) understanding the biology of oocysts sporulation in Eimeria and related genus, such as Cyclospora, b) discovery of new biomarkers that can be used for determination of viability, and developmental stages, such as sporulated and unsporulated stages, in these organisms.

The manuscript is well written, and well organized.

*Thank you. We are gratified to know that our efforts at clarity succeeded.*

Major comments:

- Authors do not explain why the first 24 h of sporulation process is analyzed in this study. The question here is that; does the first 24 hour cover the whole sporulation process for E. acervulina? Please explain in the manuscript.

*Thank you for the opportunity to elaborate. The revised text now reads:*

All *Eimeria* and *Cyclospora* species share a common life cycle: environmentally resilient immature oocysts are passed from infected hosts and undergo extrinsic development before becoming infectious. The oocysts of *Eimeria spp.* infecting chickens require oxygen and optimally sporulate at 29°C. Sporulation time varies by species, but sporulation is normally completed in 24-48 hours [10]. In *E. acervulina,* sporulation is typically complete by 22 hours [10]. Oocysts of *C. cayetanensis* may take up to a week to sporulate [3]*.* Sporulated (mature) avian *Eimeria* oocysts, once ingested, mechanically ruptured, and stimulated, each release four sporocysts. Each sporocyst contains two sporozoites, which are released upon sporocyst excystation. Exposure to bile salts and digestive enzymes hastens this process. Motile sporozoites invade intestinal enterocytes and then undergo asexual development. Sexual development produces oocysts, which are passed into the intestinal lumen and excreted (unsporulated) with feces.

*We have also added empirical data and supplementary figures S1 and S2, illustrating the progress of sporulation under our study conditions:*

By the 24^th^ hour, 83% +/- 4% of oocysts appeared to have completed sporulation as indicated by the presence, in each, of four distinct sporocysts (S2 Figure).

- Authors state that “ At each time point, oocysts were centrifuged at 3,500 rpm for 5 min at 4°C and washed with deionized water to remove excess potassium dichromate. T0 and T24 oocysts were examined at 400X with an Axio microscope (Zeiss, Germany). Images were captured using a Zeiss AxioScope camera and AxioVision imaging software.” Line 145-149. What was the sporulation rate at the 24h time point (and at other time points, if data is available)? Also representative images at different time points should be made available as supplementary data.

*Thank you for these questions.* *As sporulation represents a continuum (an impression reinforced by our study’s transcription data), it can be difficult to classify sporulation rates at intermediate time points, when parasites are undergoing morphological change. We have included two new Supplementary figures illustrating typical microscope images at hours 0 and 24. Regrettably, we did not take images at each interval. We now report the extent of sporulation at the study’s conclusion:*

By the 24^th^ hour, 83% +/- 4% of oocysts appeared to have completed sporulation as indicated by the presence, in each, of four distinct sporocysts (S2 Figure).

- Sporulated/mature, and un-sporulated/immature oocysts terms were used interchangeably in different parts of the manuscript. I recommend to use either sporulated- un-sporulated, or immature- mature terms throughout the manuscript, unless authors assign different meaning to these terms. If so, please explain.

*We appreciate the confusion introduced by using seemingly interchangeable terms. We clarify:*

In this study, we define parasite cohorts enriched for unsporulated oocysts as immature and cohorts enriched for sporulated oocysts as mature.

*Throughout the revised text, we now adhere to immature and mature parasite cohorts, reserving the terms sporulated and unsporulated for cases when discussing the status of individual oocysts.We n*

Minor corrections & comments:
- Lines 317-318, change to “We used Transcripts Per Million (TPM), as normalization method, to summarize expression of the 6,867 annotated genes. *Inserted “a” before “normalization.”*

- Line 323, change to “-during this time interval”. *Changed.*

- Line 339, do you mean “Only ~600-900 genes (<15% of the genes) in each time point were expressed >100 TPM (Table 2)? *Yes. Changed.*

- Line 339-340, “To better understand global expression, we conducted an analysis of transcriptional bias at each time point.” Please clearly explain what you mean by “transcriptional bias”.

*We added this explanation*:

That bias reflects the variance in how much each transcript contributes to the total and is calculated as 1 minus the sum of the square of each transcript’s relative frequency.

- Line 787, Please change to “--Cyclospora may constitute—“. Cyclospora and Eimeria are separate genus under Eimeriidae family, under current scientific classification. *Changed.*

- Lines 1016-1020, “We identified four genes (EAH_00007450, EAH_00054340, EAH_00057900, and EAH_00067850) that steadily increased in expression in maturing oocysts and encode enzymes involved in major energy and metabolic pathways. These enzymes (glyceraldehyde-3-phosphate dehydrogenase, LDH, Fructose-bisphosphate aldolases, respectively)”. 4 genes, 3 enzymes, respectively. Which 3 genes encodes 3 enzymes, clarify.

*We apologize for the confusion. We now clarify:*

These enzymes (glyceraldehyde-3-phosphate dehydrogenase, LDH, and two Fructose-bisphosphate aldolases, respectively) are key components of processes that likely provide energy stores to sporozoites, including glycolysis and gluconeogenesis

- Line 1101, “…and GRAs are…” Please enter open name[dense granule antigen?/protein].

*Thank you. We have revised that line accordingly:*

Micronemes and GRAs (dense granule proteins) are part of the apical complex of organelles; proteins secreted from these organelles enable host invasion by promoting parasite motility, host cell adhesion, and host cell remodeling [80, 115]*.*

*GRAs also remain defined in the sentence beginning line 691 “A few exceptions included EAH_00002690, EAH_00007450, EAH_00017540 (a hypothetical protein matching a TRAP-like protein in Plasmodium chabaudi adami), EAH_00049210 (a hypothetical protein matching tubulin-promoting proteins from other species), EAH_00049920 (a hypothetical protein matching dense granule protein [GRA]12 in T. gondii), and EAH_00064490 (matching a SAG family member protein in E. mitis):*

- Line 1314, “…and strains of C. cayetanensis…” Please use the term “isolates” instead of “ strains”, because C. cayetanensis is uncultarable, strains cannot be defined. *Changed.*

Reviewer #2: The manuscript submitted by Tucker et al describes a detailed RNAseq time course study of sporulation in Eimeria acervulina oocysts. The work is detailed and well referenced, adding to the body of knowledge for Eimeria. Extrapolation to Cyclospora is topical and relevant.

Major comments
1. The work very clearly presents detailed transcript variation between immature (unsporulated, non-infectious) and mature (sporulated, infectious) oocysts, highlighting several candidate biomarkers for sporulation. However, detection of transcripts associated with completion of sporulation is not a clear biomarker for viability or ongoing infectious risk. What happens to these transcripts as oocysts age, deteriorate, and eventually die? The authors do eventually touch on this, but comments in prominent sections of the manuscript such as the abstract should reflect this key point.

*Thank you. We have modified the Abstract. The important caveat highlighted by the reviewer is addressed in the second-to-last sentence:*

*Eimeria* parasites cause enteric disease in livestock and the closely related *Cyclospora cayetanensis* causes human disease. Oocysts of these coccidian parasites undergo maturation (sporulation) before becoming infectious. Here, we assessed transcription in maturing oocysts of *Eimeria acervulina*, a widespread chicken parasite, predicted gene functions, and determined which of these genes also occur in *C. cayetanensis*. RNA-Sequencing yielded ~2 billion paired-end reads, 92% of which mapped to the *E. acervulina* genome. The ~6,900 annotated genes underwent temporally-coordinated patterns of gene expression. Fifty-three genes each contributed >1,000 transcripts per million (TPM) throughout the study interval, including cation-transporting ATPases, an oocyst wall protein, a palmitoyltransferase, membrane proteins, and hypothetical proteins. These genes were enriched for 285 gene ontology (GO) terms and 13 genes were ascribed to 17 KEGG pathways, defining housekeeping processes and functions important throughout sporulation. Expression differed in mature and immature oocysts for 40% (2,928) of all genes; of these, nearly two-thirds (1,843) increased their expression over time. Eight genes expressed most in immature oocysts, encoding proteins promoting oocyst maturation and development, were assigned to 37 GO terms and 5 KEGG pathways. Fifty-six genes underwent significant upregulation in mature oocysts, each contributing at least 1,000 TPM. Of these, 40 were annotated by 215 GO assignments and 9 were associated with 18 KEGG pathways, encoding products involved in respiration, carbon fixation, energy utilization, invasion, motility, and stress and detoxification responses. Sporulation orchestrates coordinated changes in the expression of many genes, most especially those governing metabolic activity. **Establishing the long-term fate of these transcripts in sporulated oocysts and in senescent and deceased oocysts will further elucidate the biology of coccidian development, and may provide tools to assay infectiousness of parasite cohorts.** Moreover, because many of these genes have homologues in *C. cayetanensis,* they may prove useful as biomarkers for risk.

*We have also modified the concluding Discussion paragraph to highlight these considerations:*

Extending such studies to sporocyst excystation and invasion will further establish biomarkers for viability, as not all mature oocyst cohorts, enriched with sporulated oocysts, are infectious. Pairing gene expression with functional assays of infection in poultry could substantially contribute to risk assessment, novel treatments, and mitigation in *C. cayetanensis,* for which no animal or cell culture propagation system yet exists**. Such applications await determination of the long-term fate of these transcripts in sporulated oocysts and in senescent and deceased oocysts.** Because many of these genes have homologues in *C. cayetanensis*, they may prove useful as biomarkers for human risk. Therefore, future studies will aim to empirically determine whether expression profiles in *E. acervulina* parallel those in human parasites.

Minor comments
1. Line 33 and below. While some transcripts are found to be expressed throughout the 24 h sampling window of oocyst maturation, I don’t think these should be called ‘constitutive’, since the rest of the lifecycle is not considered. For example, an oocyst wall protein would not be constitutively transcribed.

*Thank you. We did not mean to imply knowledge of expression at other times, for example when infecting host cells. To clarify, we have defined our use of ‘constitutive’.*

For present purposes, we designate these genes as highly constitutively-expressed during the 24-hour sporulation we studied. We do not, however, know which of these genes are transcribed throughout the entire life cycle, including those stages infecting host cells.

2. Line 63-64. The toxin producing C. perfingens strains that cause NE in chickens are not usually the same that cause disease in humans. Suggest remove text after reference [2]. *Removed.*

3. Line 81. 10% of what? - People, presumably, rather than the foodstuffs. *Yes, of people. Corrected.*

4. Lines 96-97. Sporozoite release requires stimulation - it is not inevitable after oocyst rupture. *Thank you. We have revised:*

Sporulated avian *Eimeria* oocysts, once ingested, mechanically ruptured, and stimulated, each release four sporocysts.

5. Line 136 and below. Centrifuge speeds should be given in g, not rpm, or give rotor diameter. *Changed in each instance.*

6. Line 140. What concentration(s) were the oocysts sporulated in?

Oocysts were enumerated by microscopy with a hemocytometer (yielded 10^7^-10^8^ total oocysts per replicate). The final preparation of oocysts was resuspended in 2% v/v potassium dichromate in a sterile 1-L flask and incubated in a 29°C shaking water bath.

7. Table 3. Can the authors add a column for T12? This can improve characterization per transcript.

*We have revised the table to illustrate Transcripts per million at T12 (for the originally listed genes, listed because of significant increases their expression over time).*

8. The discussion is detailed and well referenced, but it is very long with some repetition from the results - 12 pages is a very long discussion. Can this be abbreviated?

*We appreciate that the discussion is lengthy and endeavored to minimize repetition, while allowing readers to derive meaning from strategic reading of portions of greatest interest.*
